# Condylar Parameters and Mandibular Movement Patterns in Bruxers Using an Optical Jaw Tracking System

**DOI:** 10.3390/jcm13247761

**Published:** 2024-12-19

**Authors:** Manuela Tăut, Solene Chanteux, Andreea Kui, Rareș Buduru, Marius Negucioiu, Manuela Manziuc, Ioana Gheorghiu, Mihaela Hedeșiu, Smaranda Buduru, Aranka Ilea

**Affiliations:** 1 Department of Oral Rehabilitation, “Iuliu Hațieganu” University of Medicine and Pharmacy, 400029 Cluj-Napoca, Romania; manuela.taut@elearn.umfcluj.ro (M.T.);; 2 Department of Prosthetic Dentistry and Dental Materials Department, Iuliu Hațieganu University of Medicine and Pharmacy, 32 Clinicilor Street, 400006 Cluj-Napoca, Romania; 3 Graduate of Faculty of Dental Medicine, University of Medicine and Pharmacy “Iuliu Hațieganu”—Cluj-Napoca, 400029 Cluj-Napoca, Romania; 4 Department of Oral Surgery, Stomestet Dental Clinic, 400515 Cluj-Napoca, Romania; 5 Department of Medical Education, Medical Informatics and Biostatistics, “Iuliu Hatieganu” University of Medicine and Pharmacy, 400029 Cluj-Napoca, Romania; 6 Department of Maxillo-Facial Surgery and Radiology, Dental Radiology, “Iuliu Hațieganu” University of Medicine and Pharmacy, 400029 Cluj-Napoca, Romania

**Keywords:** bruxism assessment, biomechanics, condylar kinematics, jaw tracking, digital dentistry

## Abstract

**Background/Objectives**: Eccentric bruxism is a complex parafunctional activity that involves grinding of teeth and occurs more frequently during sleep. This study aimed to assess differences in condylar parameters (sagittal condylar inclination -SCI and Bennett angle -BA) and mandibular and condylar kinematics during functional and parafunctional movements in bruxers and non-bruxers and to assess a digital method for quantifying eccentric bruxism using an optical jaw tracking system (Modjaw^®^). **Methods**: The study group included subjects diagnosed with eccentric bruxism according to validated clinical diagnostic criteria. A control group of non-bruxer subjects with demographic characteristics similar to the study group was considered. Each participant underwent Modjaw^®^ examination twice to assess the recordings’ repeatability. The anterior guidance, mastication, and simulated eccentric bruxism were recorded. The SCI and BA were computed. The trajectories of interincisal inferior point (IIP), left condyle (LC), and right condyle (RC) in the frontal (F), sagittal (S), and horizontal (H) planes were outlined in rectangles to calculate areas of mastication and areas of eccentric bruxism (mm^2^). Intraclass correlation coefficient (ICC) was used to assess the recordings’ repeatability. Comparisons between groups were performed using Student’s t- and Mann–Whitney tests. The receiver–operator characteristic (ROC) curve was used to assess the diagnostic quality of the digital method. **Results:** Twenty bruxers (10 F and 10 M) and 20 non-bruxers (10 F and 10 M) were included. The ICC had values higher than 0.85. SCI, BA, and area of mastication for IIP, LC, and RC were similar between the groups (*p* > 0.05). The area of eccentric bruxism was significantly wider in the bruxers (*p* < 0.001). According to the ROC curve, the following cut-off areas (mm^2^) for eccentric bruxism were found in F, S, and H planes: IIP (18.05, 13.43, 16.28); LC (3.74, 10.83, 3.35); and RC (4.21, 10.63, 2.9), corresponding to sensitivity > 0.8, specificity > 0.75 and area under the curve (AUC) > 0.85. **Conclusions:** Mandibular and condylar kinematics during functional movements were similar in bruxers and non-bruxers. A novel digital method for quantifying eccentric bruxism was found using Modjaw^®^, which could serve as a tool for early detection of eccentric bruxism before the onset of clinical consequences.

## 1. Introduction

The consensual definition of bruxism is described as “the parafunctional grinding of teeth and an oral habit consisting of involuntary rhythmic or spasmodic nonfunctional clenching of teeth, in other than chewing movements of the mandible, which may lead to occlusal trauma” [1].

According to the circadian cycle, bruxism can be classified into two main categories: awake bruxism, which is characterized by tonic and continuous contractions of the masseter and temporal muscles during wakefulness [2]; sleep bruxism is characterized by masticatory muscular hyperactivity during sleep, which can be either rhythmic (phasic) or non-rhythmic (tonic) [3]. The complex origins of sleep bruxism, influenced by both neurological and psychological factors, demand the development of innovative diagnostic methods [4,5].

Another classification is based on clinical assessment of the patient during bruxism: the first type, centric bruxism, involves teeth clenching with isometric contraction of elevator muscles without any lateral movement of the jaw [6]; the second type, eccentric bruxism, involves lateral movements of the jaw and is characterized by phasic contractions of masseter muscles observed on electromyographic devices and repetitive or prolonged teeth grinding. Grinding occurs more frequently during sleep [7].

According to Manfredini et al. [8], a comprehensive assessment of bruxism should be conducted. Hence, the Standardized Tool for the Assessment of Bruxism (STAB) was proposed consisting of two distinct primary axes. Axis A consists of self-reported patient data on bruxism and potential repercussions (i.e., behaviors of clenching, grinding, teeth contact, bracing of the mandible, jaw or muscle symptom, headache, xerostomia, tinnitus), clinical assessment (i.e., temporo-mandibular joint—TMJ and elevator muscles evaluation) and instrumental assessment (data collected from technological devices) [8]. Axis B refers to the assessment of comorbid diseases and etiological and risk factors for bruxism [8].

According to a systematic review and meta-analysis conducted by Zieliński et al. [9] the global bruxism (sleep and awake) prevalence is 22.22%, with significant variation across continents: sleep bruxism ranges from 19% in Asia to 21% in Europe, respectively and 29% in North America; awake bruxism ranges from 18% in Europe to 30% in South America. Polysomnographic studies provide a more detailed view of sleep bruxism, estimating its prevalence at 43%.

Polysomnography (PSG) is the gold standard for diagnosing eccentric mandibular movements and sleep bruxism [10]. Other techniques were described for the assessment of eccentric bruxism: sensors-equipped oral appliances, smartphone or smartwatch applications, and portable electromyographic recording devices [11,12].

Consideration must be given to the biomechanical consequences of bruxism on the TMJ and masticatory muscles [13]. Several systematic reviews [14,15] investigated the association between bruxism and muscle pathology (myalgia) or intra-articular pathology (arthralgia, joint sounds or osseous degeneration). The parameters that are used to describe the TMJ’s structural integrity are the shape and inclination of the articular eminentia (the sagittal condylar inclination—SCI and the Bennett angle—BA) [16]. SCI and BA are accurately assessed on cone beam computed tomography (CBCT) [17]. Moreover, other digital devices were described to assess qualitative and quantitative data regarding condylar and mandibular kinematics: Modjaw^®^ (Modjaw, Villeurbanne, France) [18], Zebris Jaw Motion Analyzer+ Optic System (Zebris Medical GmbH, Isny, Germany) [19] and Cadiax Compact^®^ II condylography system (Gamma Dental, Klosterneuburg, Austria) [20].

MODJAW^®^ combines the morphological characteristics of the TMJ with mandibular motion to create the first JAW Morphodynamics data through optical jaw tracking systems [21]. Modjaw^®^ jaw tracking system can record the patient’s envelope of function, functional or parafunctional movements and static maxillomandibular relationship at centric relation position (CR) [22].

Despite the advancements in digital technologies, the integration of the optical jaw tracking systems for the assessment of functional and parafunctional condylar and mandibular kinematics is sparse [18,19,21]. Given the limited data on mandibular movement patterns and the established positive correlation between the development of temporo-mandibular disorders (TMD) and both awake and sleep bruxism [14], further investigation into these patterns is crucial to advancing our understanding of bruxism’s impact on TMD and dental health. Subsequently, the study of these motions remains a field open to explore.

The objectives of this in vivo case-control study were:

1. To compare the SCI and BA at 5 mm condylar displacement between bruxer group and the control group during protrusive and laterotrusive movements using Modjaw^®^;
2. To compare the area of mastication and the area of eccentric bruxism for between the bruxer group and control group during mastication and simulated eccentric bruxism using Modjaw^®^;
3. To assess the diagnostic quality of a digital method for quantifying eccentric bruxism using Modjaw^®^.

The first null hypotesis was that SCI and BA at 5 mm condylar displacement are significantly different between bruxers and controls. The second null hypotesis was that bruxers exhibit larger areas of movement for mastication and eccentric bruxism. The third null hypotesis was that Modjaw^®^ has a good diagnostic quality for the assessment of eccentric bruxism.

## 2. Materials and Methods

### 2.1. Study Design

A prospective case-control study was carried out in November 2024 at the “Iuliu Hatieganu” University of Medicine and Pharmacy in Cluj-Napoca, Romania (Research Ethics Committee no. 211/06.11.2024). The study was conducted following the Declaration of Helsinki, and all participants completed and signed the informed consent. Each participant underwent a comprehensive clinical examination for the assessment of bruxism [23,24]. Modjaw^®^ examinations were conducted after being enrolled in this study.

### 2.2. Participants

The inclusion criteria for the study group were as follows: (1) self-reported symptoms of bruxism: headaches or difficulties in mouth opening in the morning, soreness or tenderness in the masticatory muscles; (2) symptoms of bruxism confirmed by a sleep partner: teeth grinding; (3) age: 18 to 70 years old; (4) signs of eccentric bruxism: horizontal tooth wear, hypertonia or hypertrophy of masseter muscles; (5) complete natural dentition except for the third molar; (6) voluntary participation: willingness to participate in the study and undergo all diagnostic procedures as outlined in the study protocol. The exclusion criteria were: (1) existing dental or jaw disorders: clinically diagnosed intra-articular TMD (arthralgia, click or popping, crepitus), severe malocclusion, ongoing orthodontic treatment, or extensive dental restorations; (2) unilateral chewing habits; (3) dental erosion; (4) chewing habits that may alter the masticatory muscles (e.g., habitual gum chewing); (5) neurological conditions: Parkinson’s disease or history of strokes; (6) use of medications: muscle relaxants, sleep medication, or psychoactive drugs; (7) diagnosed psychological disorders.

A control group of healthy asymptomatic subjects without any previously orthodontics, prosthodontics, or occlusal splint therapy, presenting similar demographic characteristic participants (age and gender) as the study group, and willing to participate was also included.

A systematic clinical evaluation was conducted by a calibrated, experienced examiner (S.B.) based on the validated clinical diagnostic criteria for eccentric bruxism for both the study group and the control group. According to American Sleep Disorders Association [23] and and revised by the American Academy of Sleep Medicine [24], a positive clinical diagnostic of eccentric bruxism was indicated if: (1) a positive history of tooth grinding for at least three nights per week for the last six months, as confirmed by a sleep partner; (2) clinical signs of tooth wear; (3) hypertrophy of masseter muscle; and (4) self-reported tenderness or muscle fatigue in the morning. Additionally, a clinical evaluation form for bruxism was completed for each patient by the same examiner resulting in BRUXIex index score [25] (Table S1, taut supplementary). Moreover, unimanual manipulation technique for CR [26], TMJ and muscle examination according to research diagnostic criteria for TMD (RDC/TMD) [27] were conducted by the same examiner for both the study group and the control group.

### 2.3. Modjaw^®^ Examination

Two separate Modjaw^®^ registration sessions were conducted by the same experienced examiner (M.T.) for each patient, one week apart, to guarantee accurate data acquisition and to assess the recordings’ repeatability. To minimize the effects of muscle fatigue during each Modjaw^®^ examinations, every motion was performed with a one-minute break between repetitions. The optical impressions were taken using a Trios3 intraoral scanner (IOS) (3Shape A/S, Copenhagen, Denmark). Then, the stereolithography files (.STL) were uploaded into Modjaw^®^ TWIM software to be positioned correctly according to reference planes (axio-orbitar plane and mediosagittal plane). The Modjaw^®^ examination started with the unimanual manipulation in CR and the software displayed the condylar and mandibular kinematics. If the condyles’ amplitude was limited to 1 mm^2^ and the trajectory of the interincisal inferior point was straight in frontal plane, without deviation, the software computed automatically the condyles’ real hinge axis.

Three protrusive and laterotrusive movements were recorded in each session. To ensure that the movements would be carried out correctly, the patient was trained to do protrusive and laterotrusive movements prior to data recording. The mastication recording started with a calibrated apple slice of 1 cm^2^ between the tongue and palate. Subjects were told to masticate freely on both sides until swallowing. Finally, to simulate eccentric bruxism movements, subjects were asked to grind freely their teeth forward–backward and right–left for 10 s.

### 2.4. Outcome Measurements

The real hinge axis of condyles was validated from the recording of the centric relation and computed before analyzing the quantitative data. Hence, for every movement that was recorded, the software automatically adjusted the condylar displacements around the real hinge axis as a starting position to assess the condylar kinematics.

**Figure 1:**
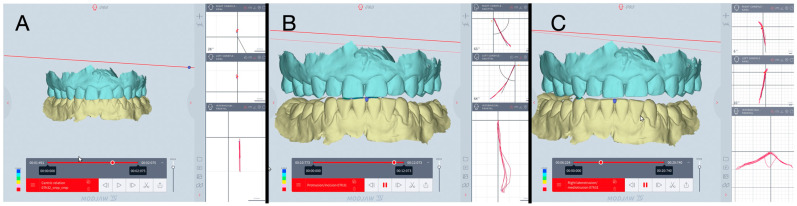
Modjaw^®^ examination: (**A**). real hinge axis computation; (**B**). SCI—computation; (**C**). BA—computation.

In each session, three successive protrusive movements were displayed, and the right and left SCI were automatically computed at 5 mm condylar displacement in the sagittal plane. Three successive laterotrusive movements were displayed, and the right and left BA on the opposite side were automatically computed at a 5 mm condylar displacement in the axial (horizontal) plane (Figure 1).

The recordings’ repeatability was assessed for SCI and BA.

The kinematics of the mandible and condyles were assessed during mastication and simulated eccentric bruxism by analyzing the trajectories of the interincisal inferior point (IIP), LC (left condyle) and RC (right condyle) in the frontal (F), sagittal (S) and horizontal (H) planes. Trajectory refers to the specific path (continuous line or curve) traced by the mandible and condyles during motion. For mastication, the trajectory displayed prior to swallowing was analyzed. For simulated eccentric bruxism, the trajectory displayed for the first 10 s was analyzed.

**Figure 2:**
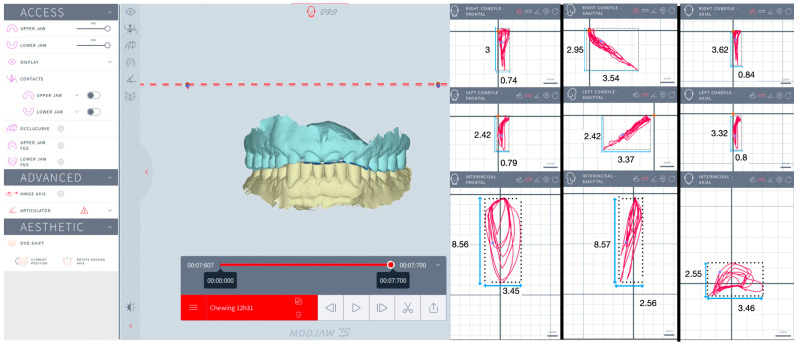
Mastication for a bruxer: interincisal inferior point, left condyle, and right condyle in the frontal, sagittal, and horizontal planes.

**Figure 3:**
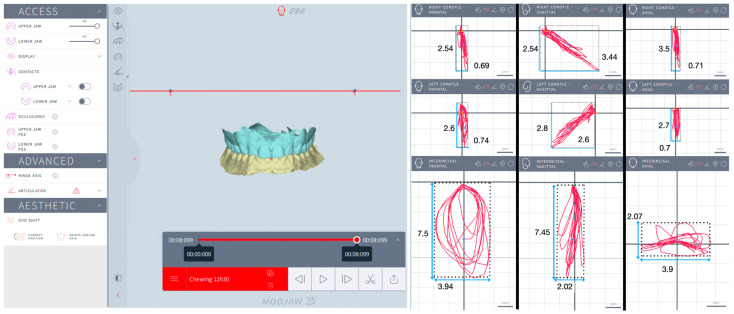
Mastication for a non-bruxer: interincisal inferior point, left condyle, and right condyle in the frontal, sagittal, and horizontal planes.

**Figure 4:**
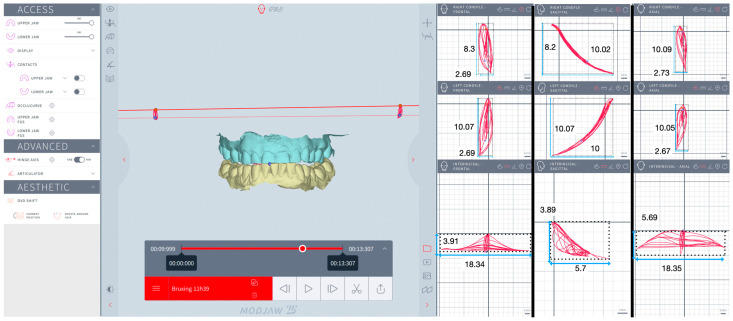
Simulated eccentric bruxism for a bruxer: interincisal inferior point, left condyle, and right condyle in frontal, sagittal, and horizontal planes.

**Figure 5:**
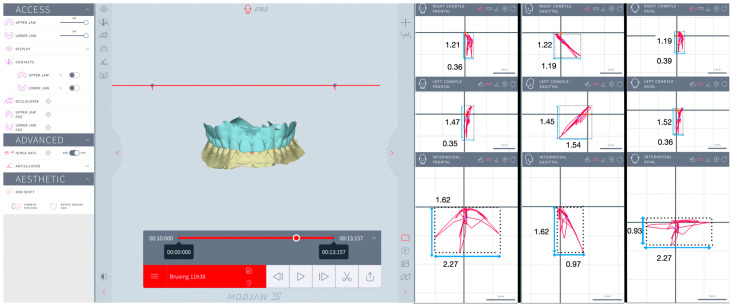
Simulated eccentric bruxism for a non-bruxer: interincisal inferior point, left condyle, and right condyle in frontal, sagittal, and horizontal planes.

The recorded trajectories of the IIP, LC, and RC were visualized in three spatial planes. The F plane displayed the vertical and lateral movement, the S plane displayed the vertical and anteroposterior movement, and the H plane displayed the lateral and anteroposterior movement. In each plane, four extreme points of the trajectory were identified, and tangents to these points were drawn parallel to a vertical plane and to a horizontal plane, respectively. Thus, rectangles outlining the trajectories were obtained to measure the height and width for IIP, LC, and RC in three spatial planes. In the F plane, the height was measured as the distance between the highest and lowest points on the trajectory, and the width was measured as the distance between the farthest left and right points on the trajectory. In the S plane, the height was measured as the distance between the highest and lowest points on the trajectory, and the width was measured as the distance between the farthest forward and backward points on the trajectory. In the H plane, the height was measured as the distance between the farthest forward and backward points on the trajectory, and the width was measured as the distance between the farthest left and right points on the trajectory. The Modjaw^®^ interface allowed zooming in as much as needed to ensure accurate identification of the extreme points, thereby improving the reliability of the measurements (Figure 2, Figure 3, Figure 4 and Figure 5).

One independent examiner (R.B.) carried out all the measurements in the same session: height and width for IIP, LC, RC in the F, S, and H planes for each subject, for each session. The area (mm^2^) of mastication and eccentric bruxism for IIP, LC, and RC in the F, S, and H planes was calculated by multiplying the height and width for each subject, for each session. The mean values for the area (mm^2^) of mastication and eccentric bruxism were calculated.

### 2.5. Statistical Analysis

IBM SPSS Statistics (version 28), Statistica StatSoft OK (version v.13.5) and R software v.4.1.1 [28] were used for the statistical analysis based on mean values of three measurements for SCI and BA and mean values of two measurements for mastication and eccentric bruxism. The normal distribution of the data was checked visually via quantile–quantile (QQ) plots and the Shapiro–Wilk test. The intraclass correlations coefficients (ICC) were used to assess the recordings’ intra-rater reliability. The mean and standard deviations for regularly distributed data were computed, respectively, medians and lower quartile (Q1, 25%) and upper quartile 3 (Q3, 75%) for irregularly distributed data. The following statistical tests for two independent groups were used to assess the group differences: Student’s t-test for normal distributed data, Mann–Whitney U test for non-normal distributed data, and Chi-square test for nominal data. The significance threshold was set at *p* < 0.05 with 95% confidence intervals. For each statistically significant comparison we expressed the effect size for the Mann–Whitney U test as the rank–biserial correlation coefficient computed as follows: R_rb_ = 1 − (2U)/(n1 ∗ n2), where U is the Mann–Whitney statistic and n is the sample size of the groups, as reported by Kerby et al. [29]. For the interpretation of the coefficient, we followed the Colton’s criteria intervals: (0; 0.25)—no effect size, (0.25; 0.50)—small effect size, (0.50; 0.75)—moderate effect size and (0.75;1)—strong effect size [30]. Spearman rank correlation coefficients were used to test correlations between the BRUXIex index and the area of eccentric bruxism. Correlation is significant at the 0.01 level, 2-tailed *p*-value. Receiver–operator characteristic (ROC) curve analysis was carried out to test the diagnostic quality of this digital method for quantifying eccentric bruxism activity. Hence, the following quality measures were determined: the area under the curve (AUC); sensitivity; specificity; Youden index (based on formula J = sensitivity + 1-specificity); and the corresponding cut-off mandibular and condylar areas. Cut-off values were calculated based on Youden’s J statistics [31].

## 3. Results

### 3.1. Flow of Participants

For this case-control study, there were 26 subjects diagnosed with eccentric bruxism according to validated clinical diagnostic criteria. Six subjects were excluded because manipulation in CR position was not possible or because they had a positive diagnosis of intra-articular TMD according to the RDC/TMD. Hence, 20 subjects (N = 20) were included in the bruxers group. Among subjects, 10 were females (50%) and 10 males (50%), with a mean age of 32.7 ± 8.3 years.

The control group consisted of 20 subjects (N = 20). For all participants, the clinical diagnosis of eccentric bruxism was negative, a free manipulation of CR was permitted, and the RDC/TMD diagnosis was negative. There were 10 females (50%) and 10 males (50%), with a mean age of 31.05 ± 8.3 years.

Similar demographic characteristics were observed between the two groups using the Student’s t-test (*p* = 0.99) and the Chi-square test (*p* = 1.00).

### 3.2. Intraclass Correlations

The ICC intra-rater reliability had values over 0.85, showing good to excellent intraclass reliability for SCI and BA (Table 1).

### 3.3. Modjaw^®^ and Mandibular Kinematics

Table 2 summarizes the right and left SCI and BA for the study group and control group and the results of the statistical tests. No significant difference was found for the right and left SCI between the two groups (*p* = 0.58 and *p* = 0.26, respectively). Similarly, no significant difference was found for right BA and left BA between the two groups (*p* = 0.2 and *p* = 0.89, respectively).

The areas of mastication are listed in Table 3, as well as the results of the statistical tests. No significant difference was observed between groups for IIP, LC, and RC in the F, S. and H planes (*p* > 0.05).

Table 4 summarizes the areas of eccentric bruxism, as well as the results of the statistical tests. The area of eccentric bruxism was significantly higher in the study group compared to the control group for IIP, LC, and RC in the F, S, and H planes (*p* < 0.0001). A strong effect size for the Mann–Whitney U test was found for IIP in the S and H, for LC in the S and H, respectively for RC in S plane. A moderate effect size was found for IIP and LC in the F, respectively for RC in F and H planes.

Spearman rank correlation coefficients showed no significant correlation between the BRUXIex index score and the area of eccentric bruxism for both groups (Table 5).

**Figure 6:**
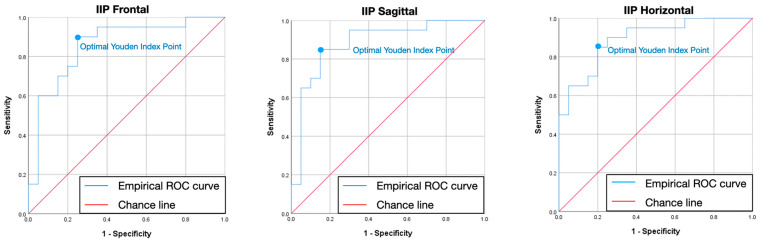
Receiver–operator characteristic curve for interincisal inferior point in frontal, sagittal, and horizontal plane. The blue dot represents the optimal sensitivity to specificity ratio (Youden index) and corresponds to the ideal cut-off value for the area of eccentric bruxism. ROC—receiver–operator characteristic; IIP—interincisal inferior point.

**Figure 7:**
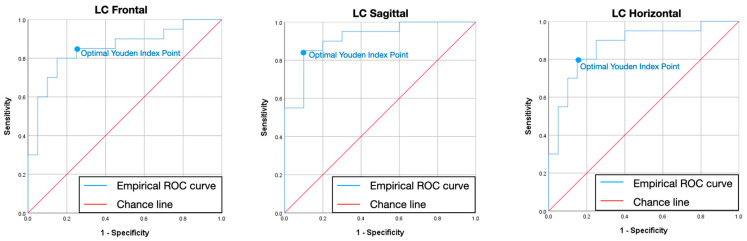
Receiver–operator characteristic curve for left condyle in frontal, sagittal, and horizontal plane. The blue dot represents the optimal sensitivity to specificity ratio (Youden index) and corresponds to the ideal cut-off value for the area of eccentric bruxism. ROC—receiver–operator characteristic; LC—left condyle.

**Figure 8:**
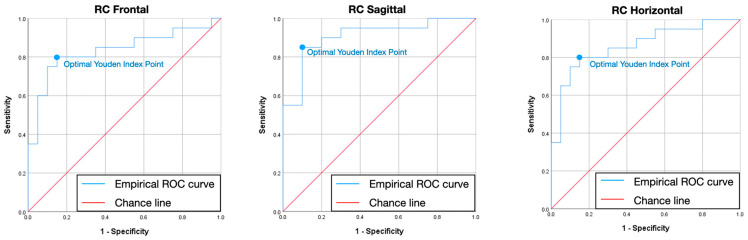
Receiver–operator characteristic curve for right condyle in frontal, sagittal, and horizontal plane. The blue dot represents the optimal sensitivity to specificity ratio (Youden index) and corresponds to the ideal cut-off value for the area of eccentric bruxism. ROC—receiver–operator characteristic; RC—right condyle.

Table 6 and Figure 6, Figure 7 and Figure 8 describe the empirically obtained ROC curve using the BRUXi index-based diagnosis (bruxer vs. non-bruxer) as a binary outcome and the area of eccentric bruxism as a predictor. The AUC corresponded to values higher than 0.85 for IIP, LC, and RC in the F, S, and H planes. Afterwards, the cut-off values for the area of eccentric bruxism were found to be ideal for IIP, LC, and RC in the F, S, and H planes, corresponding to sensitivity higher than 80% and specificity higher than 75%.

## 4. Discussion

The first null hypothesis was rejected since no significant differences were observed in SCI and BA values between the study and control groups measured from CR position to 5 mm condylar translation (*p* > 0.05). Similar values for SCI and BA at 5 mm condylar displacement were found by Nigam et al. [32] on 15 asymptomatic volunteers aged between 25 and 40 years using Modjaw^®^. Moreover, Bapelle et al. [21] found mean SCI values of 51.07 ± 9.43° during protrusion on 22 asymptomatic volunteers using Modjaw^®^. On the contrary, the mean BA value was 7.1 ± 5.1°, smaller than in our study. Nevertheless, the literature generally reported an average BA of up to 15° for healthy subjects [16,33].

From our study’s findings, it may be inferred that the osseous integrity of the TMJs was preserved in bruxers according to SCI and BA values. The absence of osseous alterations in TMJs could be attributed to a higher adaptability associated with young group age (32.7 ± 8.3 years old). This suggests that further research involving larger age groups should be considered to identify potential TMJ structural changes in bruxers. However, finding older individuals with advanced dental wear presenting no dental reconstructions could represent a challenge. Moreover, more research should be done to compare condylar characteristics measured by digital devices with those assessed by cone-beam computed tomography (CBCT) to verify potential changes in condylar structure in bruxers. However, future studies integrating dynamic three-dimensional imaging should carefully balance the benefits of enhanced diagnostic accuracy with the need to minimize risks to radiation exposure. Several systematic reviews and clinical studies have evaluated the association between bruxism and structural alterations of TMJ with mixed conclusions [34,35,36]. According to Ciancaglini et al. [34], there is no sufficient evidence to support a correlation between bruxism activity and TMD (myofascial pain, arthralgia, and disc displacement or joint degeneration). On the contrary, Manfredi et al. [35] and Jiménez-Silva et al. [36] concluded that clinically diagnosed bruxism showed a plausible association with both pain-related TMD and intra-articular TMD, including morphological changes and degenerative alterations. Furthermore, a systematic review and meta-analysis by Mortazavi et al. [14] found that sleep bruxism increased the risk of TMD by 2.06 times (odd ratio = 2.06, 95% CI: 1.82–2.30), whereas the presence of awake bruxism increased the likelihood of developing TMD by 2.51 times (odd ratio = 2.51, 95% CI: 2.02–2.99). This study demonstrated a strong positive correlation between the development of TMD and both awake and sleep bruxism. Using a novel computer simulator of loading the TMJ disc during dynamic 3D simulated tooth grinding, Sagl et al. [37] concluded that wear patterns and tooth-altered morphology may affect TMJ osseous components by loading in bruxers.

Two separate Modjaw^®^ recording sessions were conducted one week apart to evaluate the reliability of the recordings in our study for SCI and BA. All measurements showed good to excellent repeatability (ICC higher than 0.85). The real hinge axis computed according to CR unimanual manipulation was used as a starting position to assess condylar kinematics. Bapelle et al. [21] found good to excellent reliability of the Modjaw^®^ device during protrusive and laterotrusive movements at 5 mm of condylar displacement around the real hinge axis, which was consistent with our results. Poor reliability during laterotrusive movements was observed around arbitrary axis where the center of rotation was determined by locating the condyle’s center during palpation in front of the tragus at the opening and closing of the mouth [21]. Another study found the highest significant trueness and precision of the maxillomandibular relationship at centric relation by using the Modjaw-i700 IOS system, Modjaw-iTero IOS system, and Modjaw-Trios4 IOS system versus IOS alone (compared to conventional mounting in the articulator using facebow and CR registration using Kois deprogrammer) [38]. Thus, the real hinge axis computed using Modjaw^®^ is a reliable and consistent starting position from which the condylar kinematics can be accurately assessed.

Considering the second null hypothesis, it was partially rejected since no significant differences in the area of mastication for IIP, LC, and RC in the F, S, and H planes were observed between bruxers and non-bruxers. In a study involving 10 healthy subjects, the area of mastication for mandibular interincisor point was 43.8 ± 13.7 mm^2^ in the F plane, 11.1 ± 4.8 mm^2^ in the S plane, and 10.9 ± 8.5 mm^2^ in the H plane [39]. Using 3D electromagnetic articulography (3D-EMA AG501), Fuentes et al. [40] found for mandibular inter incisor point the following areas of mastication: 32.46 ± 25.02 mm^2^ in F plane and 8.3 ± 7.24 mm^2^ in S plane in 10 healthy volunteers. On the contrary, the area of eccentric bruxism was significantly higher in bruxers. In our investigation, the area of eccentric bruxism for IIP in the H plane was half of Posselt’s envelope found by Farfán et al. [39] using 3D electromagnetic articulography (64.64 mm^2^ versus 107.7 mm^2^). Similarly, the area of eccentric bruxism for LC and RC showed a significant wider area of movement in all planes when compared to the control group (7.04 to 32.91 mm^2^ for bruxers versus 1.5 to 4.22 mm^2^ for non-bruxers, *p* < 0.001). As explained by Flores-Orozco et al. [41], tooth wear may lead to more horizontal and less vertical lateral movements of the mandible, with wider excursions to perform, allowing the mandible and condyles to function freely around the border area. This causality theory between the presence of dental wear and the area of eccentric bruxism was tested in our study for each subject. The BRUXIex index is a frequently employed clinical indicator to quantify dental wear in bruxers [25]. No significant correlations were found between the BRUXIex index and the area of eccentric bruxism in F, S, and H planes for IIP, LC, and RC, suggesting that the ability to perform involuntary extensive mandibular movements is not related to the presence of dental wear both in bruxers and non-bruxers. Nevertheless, future is needed to provide additional insights into the potential interplay between movement patterns, dental wear, and eccentric bruxism.

These findings could indicate that the neural pathways and muscle memory developed throughout eccentric bruxism at night may reproduce the same pattern of movements during the day, independent of the extent or even the presence of dental wear. Since 2018, the International Community of Bruxism experts has defined sleep bruxism as “a masticatory muscle activity during sleep that is characterized as rhythmic or phasic and is not a movement disorder or a sleep disorder in otherwise healthy individuals” [42], reflecting the current paradigm shift toward a particular muscle behavior in bruxism. Additionally, the revised definition put more emphasis on the masticatory muscles’ function as the source of possible clinical consequences [42]. Palinkas et al. [43] demonstrated that sleep bruxism negatively alters the functions of masticatory muscles, supporting our findings of altered and wider mandibular movement patterns in bruxers. Câmara-Souza et al. [44] also reported differences in masticatory muscle thickness between bruxers and non-bruxers, further emphasizing the impact of bruxism on muscle morphology and function. Additionally, further investigation needs to explore the implications of bruxism’s neuropsychological triggers as a central component and its management [45].

The third null hypothesis was accepted and a novel digital method for quantifying eccentric bruxism activity presented good to very good validity parameters. According to ROC curve, the following cut-off areas (mm^2^) for eccentric bruxism were found in the F, S, and H planes: IIP (18.05, 13.43, 16.28), LC (3.74, 10.83, 3.35), and RC (4.21, 10.63, 2.9), corresponding to sensitivity >0.8, specificity >0.75, and area under the curve (AUC) >0.85. The potential for broader clinical implementation of the Modjaw^®^ device in detecting and monitoring bruxism is significant. It could impact treatment choices and enhance preventive measures, particularly given the potential clinical implications of eccentric bruxism on teeth and restorative materials wear, tooth survival in periodontitis, cracks in posterior teeth, excessive load on dental implants and their superstructures, or even implant failure and TMJ loading [46].

The average age for the study group was 32.7 ± 8.3 years, with a range between 19 and 50 years. A systematic review [47] found a consistent peak in prevalence among subjects under 40-years old among seven included studies. The Modjaw^®^ device has the potential to be used as a non-invasive tool for detecting early kinematic changes associated with bruxism from an early age to benefit from prompt interception of tooth wear and muscular pathology. The study group included ten (50%) females and ten (50%) males. A cohort of 1101 patients showed a similar rate of 53.6% females [48]. According to a systematic review and meta-analysis [9], the prevalence of sleep bruxism among females was 11.68% (95% CI, 9.07–14.07%), while among males it was 8.48% (95% CI, 7.25–9.89%). Moreover, bruxism was a significant factor among women, respectively age has been found to play a significant role in the prevalence of sleep bruxism in women.

In distinguishing between sleep and awake bruxism, we adhered to established diagnostic criteria, incorporating both clinical assessments and self-reported symptoms. To simulate bruxism episodes, we specifically focused on eccentric bruxism, given the hypothesis that eccentric bruxism predominantly occurs during sleep [8]. Eccentric bruxism involves more dynamic and extensive mandibular movements compared to typical awake bruxism, which is often characterized by clenching without significant lateral or protrusive jaw movements [8]. Thus, our simulations were designed to replicate the conditions under that sleep bruxism typically manifests, enhancing the relevance of our findings to nighttime bruxism scenarios [49].

A limitation of these clinical findings is a simulated environment where the patient was conscious during the daytime examination. The difficulty of analyzing mandibular movements in a real nighttime scenario further complicates the ability to fully capture the authentic biomechanical and neurological processes associated with sleep bruxism. Nevertheless, future studies involving advanced technology to assess mandibular movements in real nighttime conditions could further validate the findings and address circadian behavioral variability. Moreover, even if the study primarily focused on the mechanical and movement aspects of eccentric bruxism, we also acknowledge the critical role of neural control in bruxism throughout the coordination and regulation of muscle activity by the central and peripheral nervous systems. Thus, future research aiming to explore the interaction between neural control and eccentric bruxism dynamics would provide a more comprehensive understanding of these phenomena. We could not find literature on this specific topic to calculate the sample size before starting our study. Considering the exploratory nature of our research, we aimed to start with a manageable sample size that would still provide preliminary insights and allow us to identify potential patterns. Therefore, the small sample size and young age of participants may limit the applicability of the findings. Moreover, another limitation of this study was the inability to assess the impact of age on motor patterns in bruxism due to the small sample size within each age subgroup. Future longitudinal studies tracking changes in condylar movement and mandibular patterns with larger age distributions are necessary to explore the influence of age on motor patterns in bruxism, to assess their applicability across different age demographics, respectively to validate these findings.

To our knowledge, this is the first study to provide valuable insights into the condylar kinematics during functional and parafunctional movements in bruxers. Thereby, a few novelty findings could be listed as follows:

The first study on condylar inclination in bruxers: This study revealed no changes in condylar inclination in bruxers compared to non-bruxers. These results challenge the conventional understanding of bruxism’s impact on TMJ structures and highlight the importance of age-related adaptability. Comprehensive evaluation methods, combining clinical examinations with optical jaw tracking systems, are needed to prevent TMJ structural alterations in bruxers.

The first study on condylar kinematics during mastication and eccentric movements in bruxers: This study provides preliminary insights into mandibular and condylar kinematics during functional and parafunctional movements in bruxers. It uniquely applies the Modjaw^®^ device to understand the fundamental role of the stomatognathic system—that of the mandibular and condylar complex movements.

A novel digital method with high sensitivity and specificity for quantifying eccentric bruxism: An objective digital method for quantifying bruxism activity was presented in this study, showing good to very good values for sensitivity (>0.8), specificity (>0.75), and for the area under the curve (AUC) (>0.85). The potential of optical jaw tracking systems in the early detection of bruxism makes it a valuable tool in both clinical and research settings, preventing irreversible effects such as tooth wear and restorative materials chipping. Modjaw^®^ could serve as a reliable non-invasive tool in detecting bruxism with a broader clinical implementation, impacting treatment choices and enhancing preventive measures.

## 5. Conclusions

A digital method for quantifying eccentric bruxism was presented using Modjaw^®^. Mandibular and condylar cut-off areas for eccentric bruxism were obtained with high sensitivity and specificity. The reliability of Modjaw^®^ recordings was good to excellent for condylar parameters.

The optical jaw tracking systems could serve as reliable non-invasive tools for the early detection of eccentric bruxism before the onset of clinical signs and symptoms. A wider application of the digital devices in detecting and tracking bruxism could influence the clinical approach in managing bruxism. Thus, preventive strategies for tooth wear, chipping of restorative materials and structural alterations, as well as treatment strategies for early interception could be enhanced.

Condylar inclination (SCI and BA) and mandibular and condylar kinematics during mastication were similar in bruxers and non-bruxers.

## Figures and Tables

**Table 1:** The intraclass correlation coefficients for SCI and BA values.

Variables	ICC (95% CI)	*p*-Value
SCI (°)		
Right	0.931 (0.867–0.958)	<0.001
Left	0.924 (0.891–0.963)	<0.001
BA (°)		
Right	0.869 (0.810–0.933)	<0.001
Left	0.852 (0.809–0.921)	<0.001

SCI—sagittal condylar inclination; BA—Bennett Angle, CI—confidence interval.

**Table 2:** Comparisons of SCI and BA values between the examined groups.

Variables	Study Group (n = 20)	Control Group (n = 20)	*p*-Value
SCI (°)			
Right	47.5 (39–52.5)	49 (45.5–51)	0.58 **
Left	48.6 ± 5.9	50.6 ± 5.06	0.26 *
BA (°)			
Right	9.05 ± 2.9	9.2 ± 4	0.89 *
Left	10.6 ± 3.8	9 ± 4.03	0.2 *

* T-test; ** Mann–Whitney U Test; SCI—sagittal condylar inclination; BA—Bennett Angle; variables were described as average (standard deviation—SD) for normal distributed data and as median (first quartile—Q1 and third quartile—Q3) for non-normal distributed data.

**Table 3:** Comparisons of mastication area between the examined groups.

Variables of Mastication	Study Group (n = 20)	Control Group (n = 20)	*p*-Value
IIP (mm^2^)			
Frontal	32.75 (27.75–38.07)	31.21 (22.74–40.78)	0.88 **
Sagittal	15.23 ± 6.42	14.83 ± 5.56	0.78 *
Horizontal	9.33 ± 2.82	8.92 ± 3.49	0.77 *
LC (mm^2^)			
Frontal	4.21 ± 2.55	3.02 ± 1.51	0.72 *
Sagittal	8.01 (6.79–9.52)	7.05 (5.31–9.77)	0.72 **
Horizontal	2.86 ± 1.06	2.83 ± 1.33	0.93 *
RC (mm^2^)			
Frontal	3.98 ± 2.25	2.81 ± 1.45	0.68 *
Sagittal	7.66 (6.86–9.69)	6.86 (4.94–9.34)	0.73 **
Horizontal	2.79 (2.14–3.56)	2.33 (1.75–4.09)	0.77 **

* T-test; ** Mann–Whitney U Test; IIP—interincisal inferior point; LC—left condyle; RC—right condyle; variables were described as average (standard deviation—SD) for normal distributed data and as median (first quartile—Q1 and third quartile—Q3) for non-normal distributed data.

**Table 4:** Comparisons of eccentric bruxism area between the examined groups.

Variables of Eccentric Bruxism	Study Group (n = 20)	Control Group (n = 20)	Median Differences (95% CI)	*p*-Value	Effect Size
IIP (mm^2^)					
Frontal	40.49 (24.38–55.44)	7.46 (3.38–20.95)	33.02 (16.68–44.89)	<0.0001 *	0.715
Sagittal	20.86 (13.89–39.22)	3.86 (1.74–12.38)	16.99 (9.2–27.97)	<0.0001 *	0.765
Horizontal	64.64 (19.68–102.55)	7.64 (3.09–14.96)	56.99 (16.25–92.29)	<0.0001 *	0.785
LC (mm^2^)					
Frontal	8.93 (4.75–12.14)	2.04 (0.86–4.1)	6.89 (3.43–9.73)	<0.0001 *	0.700
Sagittal	31.25 (13.41–42.4)	4.22 (1.03–8.83)	27.02 (10.28–35.99)	<0.0001 *	0.830
Horizontal	7.38 (3.57–11.85)	1.5 (0.52–2.49)	5.88 (2.51–8.55)	<0.0001 *	0.745
RC (mm^2^)					
Frontal	8.53 (5.33–11.89)	1.9 (0.95–3.99)	6.63 (3.87–9.59)	<0.0001 *	0.670
Sagittal	32.91 (12.78–42.30)	4.13 (1.77–8.42)	28.77 (9.88–36.48)	<0.0001 *	0.815
Horizontal	7.04 (3.64–12.33)	1.56 (0.48–2.29)	5.47 (2.65–10.21)	<0.0001 *	0.725

* Mann–Whitney U Test; IIP—interincisal inferior point; LC—left condyle; RC—right condyle; CI—confidence interval; variables were described as median (first quartile—Q1 and third quartile—Q3).

**Table 5:** Correlations between BRUXIex index and area of eccentric bruxism for study group and control group.

Variables of Eccentric Bruxism	Correlation Coefficient for Study Group (n = 20)	Correlation Coefficient for Control Group (n = 20)
IIP (mm^2^)		
Frontal	−0.108 *	0.021 *
Sagittal	0.033 *	0.016 *
Horizontal	−0.047 *	0.079 *
LC (mm^2^)		
Frontal	0.219 *	0.030 *
Sagittal	−0.105 *	−0.045 *
Horizontal	0.224 *	−0.091 *
RC (mm^2^)		
Frontal	0.173 *	0.033 *
Sagittal	−0.095 *	−0.058 *
Horizontal	0.196 *	−0.008 *

* Spearman rank correlation coefficients, IIP—interincisal inferior point; LC—left condyle; RC—right condyle; CI—confidence interval.

**Table 6:** Area under the curve, cut-off values, sensitivity, and specificity for eccentric bruxism.

Variables of Eccentric Bruxism	AUC	Cutt-Off Value	Sensitivity	95% CI for Sensitivity	Specificity	95% CI for Specificity
IIP (mm^2^)						
Frontal	0.858	18.05	0.9	0.73–0.98	0.75	0.58–0.83
Sagittal	0.883	13.43	0.85	0.68–0.94	0.85	0.68–0.94
Horizontal	0.893	16.28	0.85	0.67–0.95	0.8	0.62–0.91
LC (mm^2^)						
Frontal	0.850	3.74	0.85	0.67–0.95	0.75	0.57–0.85
Sagittal	0.915	10.84	0.85	0.68–0.92	0.9	0.73–0.97
Horizontal	0.873	3.35	0.8	0.62–0.9	0.85	0.67–0.95
RC (mm^2^)						
Frontal	0.853	4.21	0.8	0.62–0.9	0.85	0.67–0.95
Sagittal	0.908	10.63	0.85	0.68–0.92	0.9	0.73–0.97
Horizontal	0.863	2.90	0.8	0.63–0.88	0.85	0.71–0.98

AUC—area under the curve; IIP—interincisal inferior point; LC—left condyle; RC—right condyle; CI—confidence interval.

## Data Availability

The original contributions presented in this study are included in the article/supplementary material. Further inquiries can be directed to the corresponding author(s).

## Supplementary Materials

The following supporting information can be downloaded at: https://www.mdpi.com/article/10.3390/jcm13247761/s1, Table S1: Assessment of bruxism—Bruxiex clinical evaluation form.

## Abbreviation

STAB	Standardized Tool for the Assessment of Bruxism
TMJ	Temporo-mandibular joint
PSG	Polysomnography
SCI	Sagittal condylar inclination
BA	Bennett angle
CBCT	Cone beam computed tomography
CR	Centric relation
TMD	Temporo-mandibular disorders
RDC/TMD	Research diagnostic criteria for temporo-mandibular disorders
IOS	Intraoral scanner
STL	Stereolithography
IIP	Interincisal inferior point
LC	Left condyle
RC	Right condyle
F	Frontal
S	Sagittal
H	Horizontal
QQ	Quantile–quantile
ICC	Intraclass correlations coefficients
ROC	Receiver–operator characteristic
AUC	Area under the curve
CI	Confidence interval
CBCT	Cone-beam computed tomography
