# Supplementary material for: Condylar Parameters and Mandibular Movement Patterns in Bruxers Using an Optical Jaw Tracking System
Source: J Clin Med. 2024 Dec 19;13(24):7761. doi: 10.3390/jcm13247761 (PMC11728031; doi:10.3390/jcm13247761)
Supplement: Supplementary file 1 [file jcm-13-07761-s001.zip › jcm-3347572-supplementary.pdf]

Table S1: ASSESSMENT OF BRUXISM – BRUXIex CLINICAL evaluation form

To be completed by the examiner, circle the numbers “0” for NO, “1” for Yes slightly, “2” for moderately, “3” for Yes a lot.

1	Occlusal wear, overall index (abrasion, attrition, erosion)?	0	1	2	3
2	Attrition (bruxism)	0	1	2	3
3	Abrasion	0	1	2	3
4	Erosion (chemical)	0	1	2	3
5	Abfraction	0	1	2	3
6	Shiny wear facets	0	1	2	3
7	Hypertonia of the elevator muscles	0	1	2	3
8	Hypertrophy of the elevator muscles	0	1	2	3
9	Thick alveolar bone, alveolar exostosis	0	1	2	3
10	Gonial exostosis	0	1	2	3
11	Lingual dysfunction	0	1	2	3
12	Lingual hypertrophy	0	1	2	3
13	Teeth marks on the edges of the tongue	0	1	2	3
14	Oral breathing	0	1	2	3
15	Traces of biting, sucking (inner side of cheeks, lips)	0	1	2	3
16	Maxillo-mandibular malalignment	0	1	2	3
17	Over guidance: right canine, left canine, or incisors locking	0	1	2	3
18	Non-functional canine guidance	0	1	2	3
19	Group function of more than 2 teeth per side	0	1	2	3
20	Limitation of opening movement (<40 mm)	0	1	2	3
21	Neuropathic dyskinesia	0	1	2	3
**Total = BRUXIex**					
